# Note on the use of different approaches to determine the pore sizes of tissue engineering scaffolds: what do we measure?

**DOI:** 10.1186/s12938-018-0543-z

**Published:** 2018-08-17

**Authors:** Martin Bartoš, Tomáš Suchý, René Foltán

**Affiliations:** 1 Institute of Dental Medicine, First Faculty of Medicine, Charles University and General University Hospital in Prague, Kateřinská 32, 128 01 Prague 2, Czech Republic; 2 Department of Composites and Carbon Materials, Institute of Rock Structure and Mechanics, Academy of Sciences of the Czech Republic, Prague 8, Czech Republic; 3 Department of Mechanics, Biomechanics and Mechatronics, Faculty of Mechanical Engineering, Czech Technical University in Prague, Prague 6, Czech Republic; 4 Institute of Pathological Physiology, First Faculty of Medicine, Charles University, Prague 2, Czech Republic; 5 Institute of Anatomy, First Faculty of Medicine. Charles University, Prague 2, Czech Republic

**Keywords:** Scaffold, Porosity, Pore size, Micro-CT, SEM, Bone regeneration

## Abstract

**Background:**

Collagen-based scaffolds provide a promising option for the treatment of bone defects. One of the key parameters of such scaffolds consists of porosity, including pore size. However, to date, no agreement has been found with respect to the methodology for pore size evaluation. Since the determination of the exact pore size value is not possible, the comparison of the various methods applied is complicated. Hence, this study focuses on the comparison of two widely-used methods for the characterization of porosity—scanning electron microscopy (SEM) and micro-computed tomography (micro-CT).

**Methods:**

7 types of collagen-based composite scaffold models were prepared by means of lyophilization and collagen cross-linking. Micro-CT analysis was performed in 3D and in 2D (pore size parameters were: major diameter, mean thickness, biggest inner circle diameter and area-equivalent circle diameter). Afterwards, pore sizes were analyzed in the same specimens by an image analysis of SEM microphotographs. The results were statistically evaluated. The comparison of the various approaches to the evaluation of pore size was based on coefficients of variance and the semi-quantitative assessment of selected qualities (e.g. the potential for direct 3D analysis, whole specimen analysis, non-destructivity).

**Results:**

The pore size values differed significantly with respect to the parameters applied. Median values of pore size values were ranging from 20 to 490 µm. The SEM values were approximately 3 times higher than micro-CT 3D values for each specimen. The Mean thickness was the most advantageous micro-CT 2D approach. Coefficient of variance revealed no differences among pore size parameters (except major diameter). The semi-quantitative comparison approach presented pore size parameters in descending order with regard to the advantages thereof as follows: (1) micro-CT 3D, (2) mean thickness and SEM, (3) biggest inner circle diameter, major diameter and area equivalent circle diameter.

**Conclusion:**

The results indicated that micro-CT 3D evaluation provides the most beneficial overall approach. Micro-CT 2D analysis (mean thickness) is advantageous in terms of its time efficacy. SEM is still considered as gold standard for its widespread use and high resolution. However, exact comparison of pore size analysis in scaffold materials remains a challenge.

**Electronic supplementary material:**

The online version of this article (10.1186/s12938-018-0543-z) contains supplementary material, which is available to authorized users.

## Background

The rapidly developing field of bone tissue regeneration aims to provide for the safe and predictable treatment of bone defects resulting from a range of conditions (e.g. trauma, tumor, inflammation). In this respect, the application of biomaterials in the form of scaffolds which provide a temporary template for new bone formation and suitable conditions for tissue healing offers a promising solution [1–3]. Such scaffolds present the appropriate chemical, biological and mechanical cues for the promotion of normal cellular behavior and function [4]. Ideally, such scaffolds should be capable of performing their intended function without eliciting any undesirable local or systemic effects in the host over the long term. There are various methods for scaffold fabrication (e.g. freeze-drying, solvent casting, electrospinning, rapid prototyping) leading to different 3D structure [5, 6].

Porosity, permeability and the mechanical properties of the scaffold represent crucial parameters in terms of cell ingrowth, cell growth and migration, and scaffold colonization. Scaffold porosity is determined by closed and open pores of varying size, shape, spatial distribution and mutual interconnection. Open porosity, particularly, has a substantial influence on scaffold–tissue interaction, cell migration, vascularization, mechanical properties, diffusion and fluid permeability. According to many studies [7–10] the prevalence of open porosity, a high degree of interconnection and pore sizes ranging from 100 to 300 µm is associated with a positive effect on bone tissue formation. Pores usually consist of interconnected channels rather than isolated homogeneous void spheres, which presents a major challenge with respect to pore analysis, particularly with concern to pore size. Indeed, the situation is considerably more complicated than it may at first seem as determined by 2D specimen sections showing isolated pores with circular or elliptical shapes; thus, it is advisable that pores should be evaluated with respect to their 3-dimensional structure.

Various approaches exist for the determination of scaffold porosity. For example, total porosity can be assessed using gravimetry (based on weighing the scaffold specimen and scaffold material density). Open porosity can be measured by means of liquid displacement and mercury intrusion, which is also able to provide an estimation of pore sizes [10, 11]. However, the application of these methods is inappropriate for collagen-based scaffolds since the pressure arising from mercury intrusion may alter the structure of the scaffold, or may be influenced by the swelling of the scaffold in the testing liquid. A further approach consists of SEM or optical microscopy image analysis, which allows for the direct measurement of a range of parameters (e.g. pore diameter, area and shape) in a number of 2D sections. The main advantages of this approach consist of both precise visualization at very high resolutions (in the case of SEM) and availability [12, 13]. However, the results of this 2D approach have several drawbacks, e.g. due to its being based on 2D sections, analysis is orientation-dependent, which may bias the results, especially with respect to anisotropic structures. The assessment of 3D architecture from 2D data must be subjected to a stereological approach which relies on 3D structure assumptions. Moreover, sectioning or measurement in a vacuum may result in the alteration of specimen.

A relatively novel approach for scaffold structure evaluation is micro-computed tomography (micro-CT, µCT, micro-tomography; [8, 10, 11, 14]), an X-ray-based imaging method which offers non-destructive 2D and 3D analysis in terms of structure visualization and quantification [15, 16]. Scaffolds are usually evaluated in dry state even though they are hydrated by implantation process. Hydrated scaffolds were subjected to micro-CT analysis only in a few studies [17, 18]. Modern micro-CT devices enable scanning at isotropic voxel sizes of below 1 µm. Micro-CT scanning results in a dataset of 2D projection images which are reconstructed to form cross-section image datasets. Grayscale images must be binarized prior to analysis so as to differentiate the subject from the background. This procedure is crucial and may significantly influence the results. Binarization is frequently complicated by image noise, scaffold composition (different materials with overlapping X-ray attenuation values), beam hardening and the partial volume effect, which particularly influences thin structures, resulting in their apparently lower X-ray density. Such factors may lead either to defects in the structure of the specimens analyzed (e.g. in the pore walls) or increased noise in the micro-CT images [19, 20].

This study focused on a comprehensive comparison of micro-CT and SEM analysis applied for the evaluation of the pore size of model composite collagen-based scaffolds fabricated by means of lyophilization and cross-linked under different conditions aiming at preparation of scaffolds with different 3D structure. We expected differing results from each of the parameters applied. The aims were to determine convenient parameters for the description of pore size and, generally, to compare SEM and micro-CT values and discuss the benefits and drawbacks of each approach.

## Methods

The composite scaffolds were prepared by lyophilization of a 4 wt% collagen dispersion in water (final content of collagen in scaffold was 50.5 wt%) with poly(dl-lactide) sub-micron fibers (PDLLA; 275–300 nm, 47 wt%), bovine bioapatite nanoparticles (bCaP; 2 wt%) and 0.5 wt% of sodium hyaluronate (HA) powder. The detailed preparation is described elsewhere [18, 21].

The collagen part of the composite scaffolds was cross-linked using a phosphate buffer saline solution (0.0027 M potassium chloride and 0.137 M sodium chloride, pH 7.4 at 25 °C) (PBS, Sigma Aldrich, Germany) at two different temperatures [room temperature, ~ 20 °C (“RT”) and 37 °C (“37”)] and by means of three different concentrations (maximum—“MAX”, medium—“MID”, minimum—“MIN”) of genipin (Sigma-Aldrich), namely MAX (0.67 g of genipin/1 g of collagen), MID (0.053 g/1 g) and MIN (0.026 g/1 g). The MID concentration was determined as a theoretical concentration sufficient for collagen cross-linking based on the determination of amino acid concentration in the collagen [22], namely that of lysine, hydroxylysine and arginin which are able to react with genipin by means of their free NH_2_ groups. Following a reaction period of 24 h, all the scaffolds were washed in 0.1 M Na_2_HPO_4_ (2 × 45 min) and deionized water (30 min) then frozen at − 15 °C for 5 h and finally lyophilized. A non-cross-linked scaffold was used for control purposes (original—“ORIG”). The specimens were described according to cross-linking parameters, i.e. genipin concentration and applied temperature and are presented in Table 1. 3 samples of each type were used in this study (N = 27).

**Table 1:** Summary of the scaffold specimens with parameters of the cross-linking process

Parameters of the scaffolds cross-linking		
Temperature	Genipin/collagen concentration	Abbreviation
20 °C	0.67 g/1 g	RT MAX
	0.053 g/1 g	RT MID
	0.026 g/1 g	RT MIN
37 °C	0.67 g/1 g	37 MAX
	0.053 g/1 g	37 MID
	0.026 g/1 g	37 MIN
Non-cross-linked		ORIG

### Characterization of the scaffolds by means of micro-CT analysis

The scaffold specimens were scanned using a micro-CT SkyScan 1272 (Bruker micro-CT, Kontich, Belgium) under the following parameters: pixel size 4.5 µm, source voltage 60 kV, source current 166 µA, Al filter 0.25 mm, rotation step = 0.1°, frame averaging (5), rotation 360°. The composite scaffolds were scanned in the dry state in air and mounted on specimen holders. The scanning time was approximately 4 h for each specimen. Flat-field correction was updated prior to each image acquisition.

Projection images were reconstructed to form cross-section images via NRecon software (Bruker) using a modified Feldcamp algorithm. Software correction (misalignment, ring artifact and beam hardening) was performed in order to reduce the effect of computed tomography artifacts. Visualizations were acquired using a DataViewer (2D cross-section images; Bruker) and a CTVox (3D images; Bruker). Color-coded pore size values were based on 3D structure separation analysis. Prior to structure analysis, the datasets were binarized using an adaptive threshold and despeckle operations in 3D were applied to reduce image noise. These steps were optimized using TeiGen software [23]. The volume of interest (VOI) subjected to analysis was defined by the shrink-wrap procedure in 3D. Scaffold structure analysis, including porosity analysis, was performed by means of a CTAn (Bruker). The analysis of pore size in the whole specimen was performed in 3D using a sphere-fitting algorithm. 2D sections of the scaffolds (transverse plane perpendicular to the axis of the cylindrically-shaped specimens; 5 sections) were evaluated for pore size values employing the following parameters: major diameter (MD, major diameter of analyzed pore), mean thickness (MT, based on circle-fitting algorithm similar to sphere-fitting procedure), biggest inner circle diameter (BICD, diameter of the biggest circle fitting analyzed pore) and area-equivalent circle diameter (AECD, diameter of circle of area equivalent to area of analyzed pore).

### Characterization of the scaffolds by means of SEM image analysis

The same specimens which were analyzed via micro-CT were characterized by means of scanning electron microscopy (SEM; Quanta 450 Microscope, FEI, USA) in high vacuum mode. The scaffolds were cut into 2 mm-thick discs (perpendicular to the long axis of the cylinder) prior to SEM analysis. The resulting sections were coated with a thin layer of gold in an ion sputter (Emitech K550X, Quorum Technologies, UK). The pore size dimensions were measured by means of ImageJ software (Rasband, W.S., ImageJ, US National Institutes of Health, Bethesda, Maryland, USA, http://imagej.nih.gov/ij/, 1997–2015). The manual mode of the ImageJ analyzer was used for the measurement of the average diameter of the pores. At least 40 pores were assessed at each of five SEM micrographs (mag. 100×) for each scaffold type. Randomly selected pores were analyzed for both long and short pore axis. All parameters of SEM and micro-CT methods are summarized in Table 2.

**Table 2:** Summary of methods and parameters applied in the evaluation of pore size

Characterization of the scaffolds		
Method	Parameter	Abbreviation
SEM	Average diameter of the pores	SEM
μCT-2D	Mean thickness	MT
	Major diameter	MD
	Biggest inner circle diameter	BICD
	Area-equivalent circle diameter	AECD
μCT-3D	Sphere-fitting algorithm	3D

### Statistical evaluation

The statistical analysis was performed using statistical software (STATGRAPHICS Centurion XVII, StatPoint, USA). The normality of the data was verified primarily by means of the Shapiro–Wilk and Chi Squared tests; outliers were identified via either the Grubbs’ or Dixon’s tests. Homoscedasticity was verified using the Levene’s and Bartlett’s tests. Non-parametric analysis was employed since either the assumption of normality or homoscedasticity were violated and, consequently, the Kruskal–Wallis test for multiple comparisons with a subsequent post hoc test based on the Bonferroni procedure. The Mann–Whitney W test was performed in the case of two-sample comparisons. Coefficient of variation was calculated as the ratio of the interquartile range to the median, hypothesizing that the lower the value of the coefficient of variation, the more accurate results the method gives. Statistical significance was accepted at p ≤ 0.05.

### Semi-quantitative comparison of different approaches of pore size analysis

Since exact value of pore size is not achievable, comparison of different methods based on their accuracy is complicated. For this reason, we used semi-quantitative assessment based on superiority to the other methods/parameters used in this study. Evaluated qualities were: non-destructivity, time efficacy, orientation independent direct 3D analysis, whole specimen evaluation, high resolution, irregular pore assessment, low image processing bias and widespread use. Qualities were assessed as 2 (very advantageous), 1 (advantageous) or 0 (no advantage). Total scores were assessed and compared (Table 3).

**Table 3:** Semi-quantitative evaluation of pore-size parameters comparison to each other: 2—very advantageous, 1—advantageous, 0—no advantage

Quality	Pore size parameters					
	SEM	3D	MT	MD	BICD	AECD
Non-destructivity	0	2	2	2	2	2
Time-efficacy	0	1	2	2	2	2
Direct 3D analysis	0	2	0	0	0	0
Whole specimen evaluation	0	2	1	1	1	1
High resolution	2	0	0	0	0	0
Irregular pore assessment	0	2	1	0	0	0
Image processing bias	2	0	0	0	0	0
Widespread use	2	0	0	0	0	0
Total score	6	9	6	5	5	5

## Results

### Tissue engineering scaffolds visualization

**Fig. 1:**
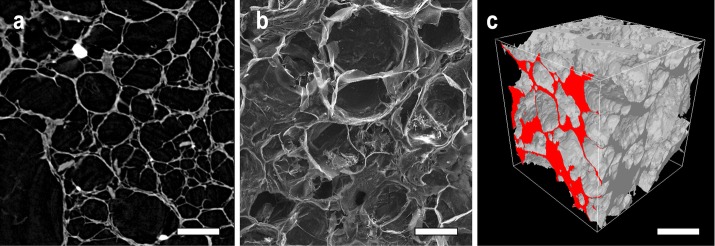
Representative images of the composite scaffold (RT MID specimen): **a** micro-CT 2D section, **b** SEM (×100), **c** micro-CT 3D image; section area is presented in red color at one side. Scale bars = 400 µm

**Fig. 2:**
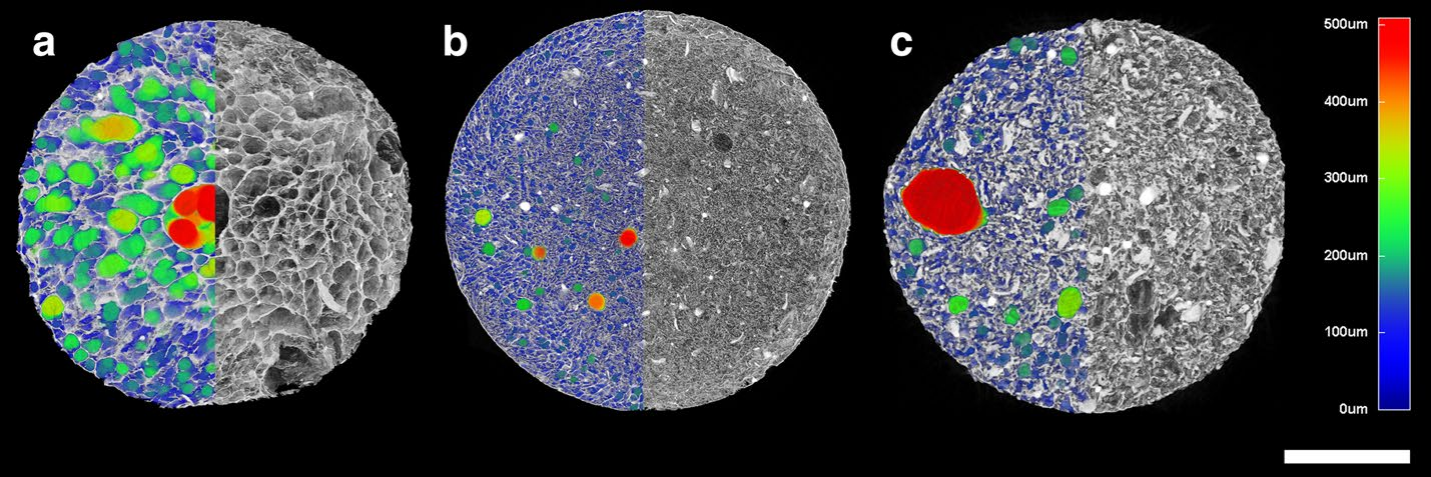
Micro-CT 3D visualization of selected specimens: **a** RT MID, **b** ORIG, **c** 37 MIN. The right halves depict the scaffold matrix, the left halves combine the scaffold matrix with color-coded pore size values. Scale bar (white) = 3 mm, the color-coded scale bar is shown on the right. The 3D dimensional structure must be considered when evaluating these images. In the ORIG (**b**) specimen red pores seem to be much smaller than red pores and even green pores in RT MID and 37 MIN. This is caused by the sectioning of the marginal part of a larger pore and can easily be misunderstood

**Fig. 3:**
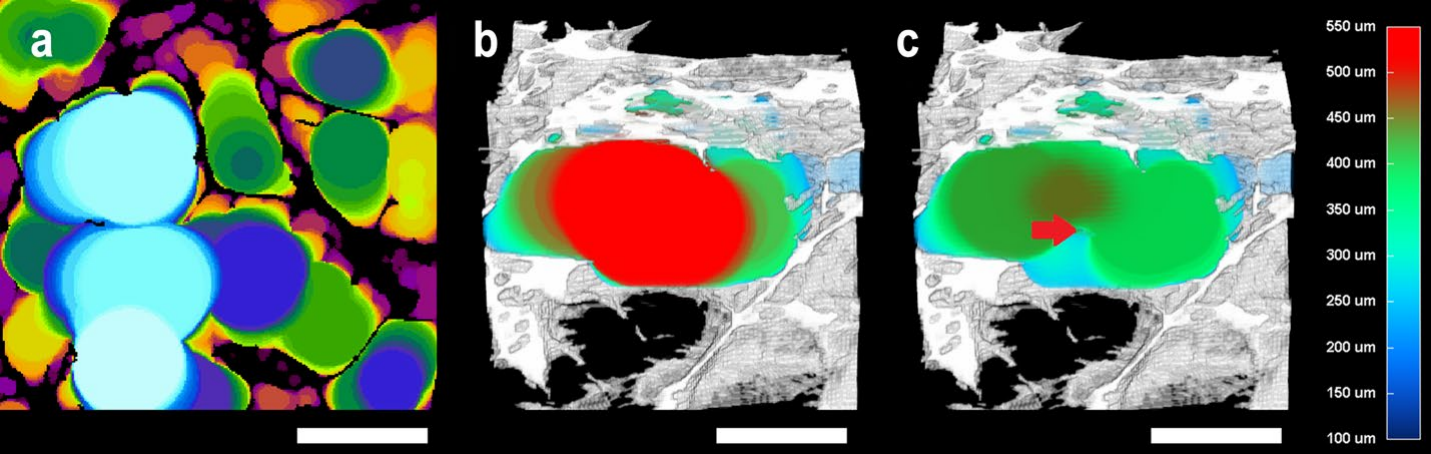
Sphere-fitting algorithm in 3D pore size evaluation and the effect of noise voxels inside a pore. **a** 2D image presenting a section through the fitted spheres inside the pores (the color is dependent on sphere diameter; the pore walls can be seen in black), **b** color-coded 3D pore size visualization of a segmented pore in 3D combined with a scaffold matrix visualization (white), **c** artificially added noise pixels are shown by the red arrow; changes in calculated pore size are apparent. The white scale bar = 500 µm; the color-coded scale bar for **b** and **c** is shown on the right; the color-coded scale bar for **a** is not shown

The structure of the scaffolds was visualized (Fig. 1) by means of SEM images and micro-CT (2D and 3D). More visualizations are presented in the Additional file 1: Appendix S1 to the study. The inner structure of the scaffolds exhibits interconnected pore spaces of various shape, pore struts and walls. Moreover, bCaP nanoparticle agglomerates are clearly visible as X-ray-dense white spots in the micro-CT images. In micro-CT thin pore walls are disconnected in some areas, indicating communication between the pores. However, this may be artificial as the result of structure thicknesses below the spatial resolution and the partial volume effect. Compared to the micro-CT 2D sections, the SEM images are not strictly limited to one section plane; thus, the evaluation of pore wall thickness and disconnections is not as accurate as initially supposed. Pore spaces of minor size in SEM are unclear and can easily be missed. 3D visualizations enable the examination of the scaffold structure from different angles, and of the inner structure by means of virtual sectioning. It is possible to combine the visualization of the scaffold matrix with pore space imaging which can be presented in color-coded mode, thus providing a comprehensive approach for the presentation of structure thickness and separation. In color-coded visualizations, we have to consider 3D dimensional structure when evaluating these images since they may seem incorrect or be misunderstood. Peripheral section of large pore may be smaller compared to medium pore sectioned in its central area, so the colors may look inappropriate (see Figs. 2 and 3). Specimens with the greatest differences in terms of pore size values (RT MID, 37 MIN) and original specimen (ORIG) were visualized by means of a color-coded 3D model (Fig. 2, Additional file 2: Appendix S3). The effect of image noise in 3D pore size evaluation is presented as color-coded visualization in Fig. 3 (further in discussion).

### Porosity quantification

**Fig. 4:**
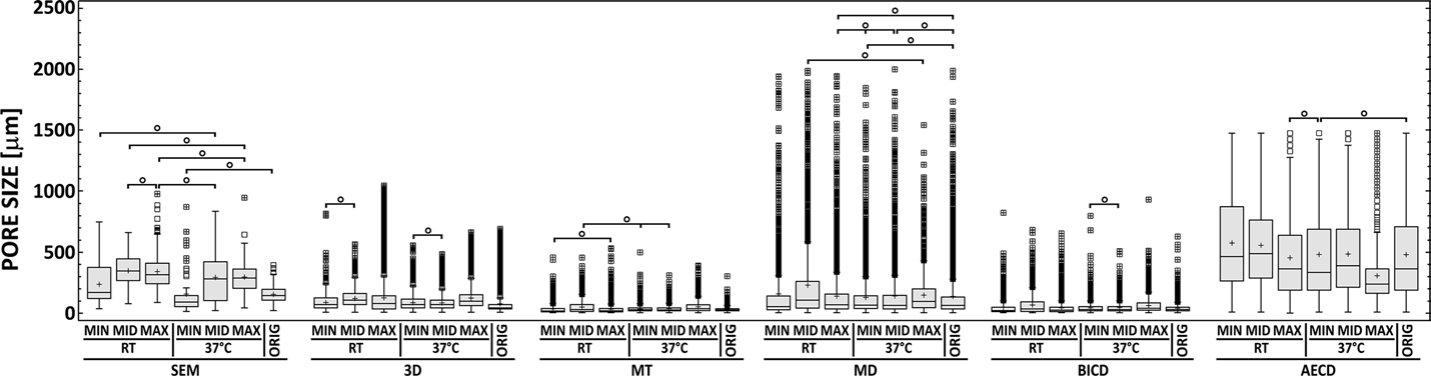
Structural porosity of differently cross-linked scaffolds expressed by means of differing 2D and 3D parameters. Open circle denotes pairs without statistically significant differences (Kruskal–Wallis, Bonferroni procedure, *0.05*)

**Fig. 5:**
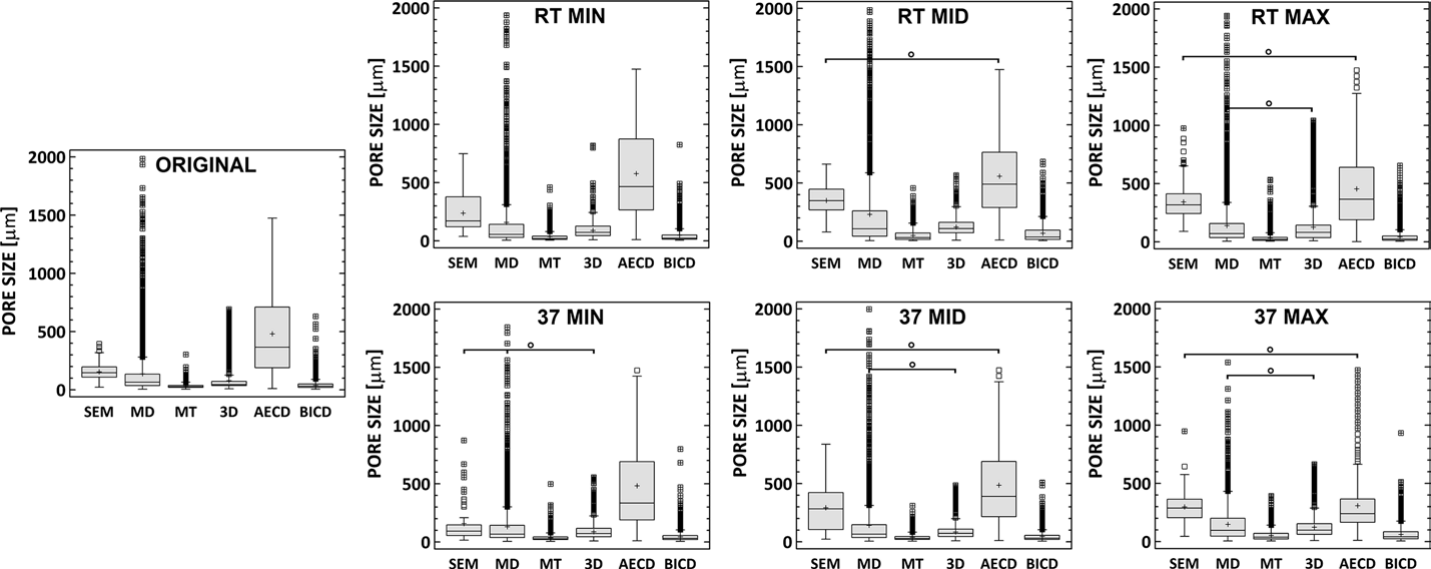
Structural porosity of differently cross-linked scaffolds expressed by means of differing 2D and 3D parameters. Statistically significant differences are evident between each of the 6 different porosity parameters of each scaffold before and after the differing cross-linking procedures except for those pairs denoted by open circle (Kruskal–Wallis, Bonferroni procedure, *0.05*)

**Fig. 6:**
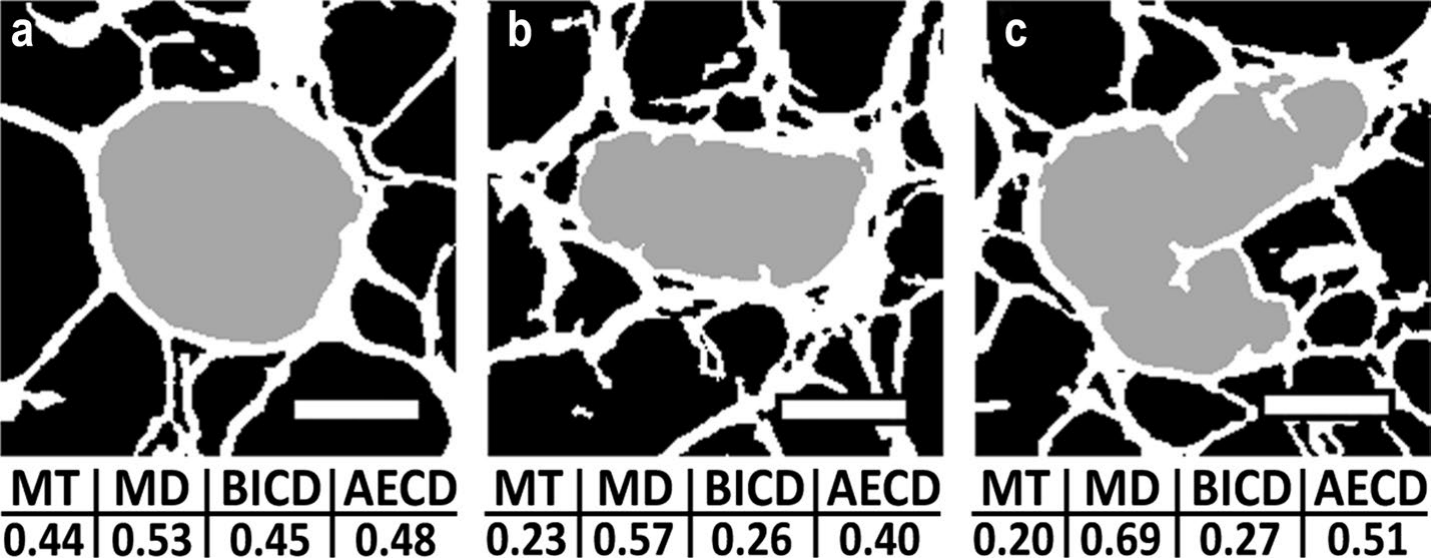
Illustration of varying results provided by micro-CT 2D pore size analysis. Pores (in gray) of 3 differing shapes (**a**, **b**, **c**) were evaluated by means of 4 micro-CT 2D parameters (MT—mean thickness, MD—major diameter, BICD—biggest inner circle diameter, AECD—area-equivalent circle diameter) and their values are presented in panels below the images (in mm). The results tend to differ with increasing shape irregularity. Scale bar = 0.2 mm

Pore sizes were evaluated in 7 model specimens using 6 different parameters (SEM, 3D, MT, MD, BICD, AECD) presenting significant differences between their values. The median, lower and upper quartile values were used for scaffold characterization purposes (Additional file 3: Appendix S2). The results are presented in Figs. 4 and 5. Median values ranged from 20 µm (MT) to 490 µm (AECD). The mean median values for each method were, in descending order: AECD (378.6 µm), SEM (235.6 µm), 3D (78.4 µm), MD (75.3 µm), BICD (31.9 µm), and MT (27.9 µm). The interquartile range was in descending order: AECD, SEM, MD, 3D, BICD and MT. The differences between the methods and parameters were statistically significant (p ≤ 0.05) with the exceptions presented in Figs. 4 and 5. Illustration of influence of pore shape in micro-CT 2D analysis is presented in Fig. 6.

### Comparison of different approaches of pore size analysis

**Fig. 7:**
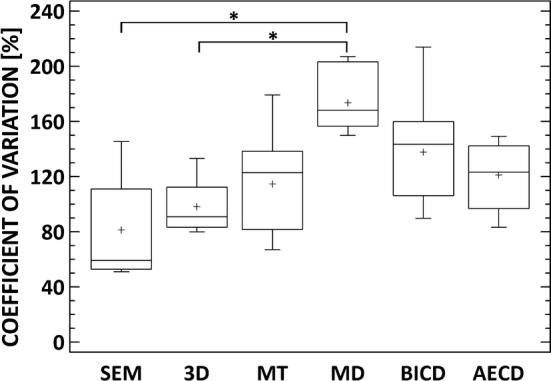
Coefficient of variation for applied method of pore size analysis. * Denotes statistically significant differences (p ≤ 0.05)

Coefficient of variation is a parameter widely used for expressing the repeatability or precision of methods in various fields, hypothesizing that the lower the value of the coefficient of variation, the more precise the measuring method or device is. The coefficient of variation is simple the ratio of the interquartile range to the median, or in other cases (e.g. in normally distributed data) the ratio of standard deviation to the mean value. Because of non-homogeneous scaffold 3D structure, pore size values present high data variance, which results to similar values of coefficient of variation in all parameters except MD (Fig. 7). Application of this approach to compare different methods is in this case limited, which is discussed below.

Semi-quantitative comparison of the parameters for pore size evaluation regarding their relative benefits is summarized in Table 3. Eight selected qualities (non-destructivity, time efficacy, orientation independent direct 3D analysis, whole specimen evaluation, high resolution, irregular pore assessment, low image processing bias and widespread use) were evaluated based on their relative advantage among the others. Total score for each parameter was calculated. Results are as follows: non-destructivity is major advantage of micro-CT; time efficacy is benefit of micro-CT, especially of 2D parameters; direct 3D analysis and whole specimen analysis is benefit of micro-CT 3D analysis (whole specimen evaluation by micro-CT 2D analysis is possible, but results require enormous further data processing); high resolution is the major advantage of SEM; evaluation of irregularly shaped pores is in principle beneficial using parameters based on averaging of fitted spheres/circles (3D, MT); possibility of important influence of image processing bias (e.g. binarization) is generally the major disadvantage of micro-CT analysis; widespread use of SEM is still superior to micro-CT to date. The highest score in descending order presents: 3D (9), MT (6), SEM (6), MD (5), BICD (5), AECD (5).

## Discussion

### Specimens, methods and parameters applied in our study

Our study focused on evaluation and comparison of pore size analysis using 2 methods (SEM and micro-CT) leading to 6 different parameters of pore size. Porosity of tissue engineering scaffolds is considered to be key structural characteristic by many authors [7, 8, 11, 24, 25]. However, there is not consent in methodology of pore size evaluation. Since 3D structure of these materials is very complex, we are generally unable to identify the exact values of its structure including porosity, which complicates comparison of different methods of pore size assessment.

To compare different pore size analysis methods, we prepared seven types of model specimens (collagen-based scaffolds intended for bone surgery application). Since 3D structure may influence the accuracy of measurement of selected parameters, we aimed at producing various 3D structure. For this reason specimens were prepared under different conditions (Table 1) which are leading to structural differences. We selected two methods (SEM and micro-CT) to evaluate pore size in our specimens. SEM was selected as a “gold standard”, micro-CT as an emerging non-destructive imaging method. With micro-CT, we evaluated pore size in 3D, which we expected to be in principle the most accurate approach. Then we selected 4 different micro-CT 2D parameters (major diameter, mean thickness, biggest inner circle diameter, area-equivalent circle diameter) in order to test whether these simple and time efficient (compared to SEM and micro-CT 3D) parameters are suitable for pore size characterization. Both positive and negative aspects of each method are further discussed below.

### SEM in pore size evaluation

SEM 2D image analysis is considered to be the “gold standard” due to its widespread use and its wide availability. The major benefit is high spatial resolution (compared to micro-CT) so thin pore walls may be better detected (see Fig. 1a, b). There are many disadvantages in comparison to micro-CT. The major disadvantage is the inability to directly assess 3D structure and limitation of the analysis to limited number of sections. Since bone-like scaffolds are not strictly isotropic, the results are dependent on the orientation of the section. SEM is based on the mechanical sectioning and special treatment of the specimen, which may result in structure alterations. This procedure is time consuming and laborious. Since SEM analysis is usually based on manual measurements, we can assume that the pores analyzed are not chosen randomly, i.e. small pores (e.g. 10–50 µm in diameter) tend to be neglected by observer, even though they may significantly contribute to the total pore area of the analyzed section. This is supported by our results (Figs. 4, 5) for SEM generally presented higher values of pore size than micro-CT (except AECD); these results may be also influenced by image noise in micro-CT analysis (more in “Micro-CT in pore size evaluation”). The exact assessment of both the pore margin and interconnection using SEM images is often difficult. The number of pores analyzed usually ranges from tens to hundreds, which is substantially less than the number achievable via automated micro-CT analysis. SEM presented the highest number of non-significant differences in pore size values in various scaffold types (Figs. 4, 5).

### Micro-CT in pore size evaluation

Micro-CT is an emerging non-destructive imaging method. There are many benefits of micro-CT application, e.g. direct 3D analysis (i.e. no orientation dependency), whole specimen evaluation and high data utilization rate, time efficacy, non-destructivity, no special treatment of specimen, easy automatization of analysis process.

However, there are many drawbacks. Analyzed virtual object based on micro-CT scans is not exactly identical to the real specimen. The partial volume effect, the structure below resolution limits and low X-ray density may result in the loss of the virtual structure. This may result in reduction of volume, changes in surfaces and higher degree of interconnection between the pores and increase in pore size [19]. Effect of binarization procedure on micro-CT results were evaluated e.g. for bone tissue [26, 27], but in collagen-based scaffolds its effect is not sufficiently described, even though is expected to be substantial. Micro-CT images are also influenced by computed-tomography artifacts, which may reduce signal to noise ratio [28]. Image noise largely influences the calculation of certain parameters of pore size based on sphere/circle fitting (in our study 3D, BICD, MT), i.e. it leads to a significant reduction in size with regard to the noise pixel 3D position inside the pore space (Fig. 3). Sensitivity to image noise differs among the parameters used in pore size description. Therefore, maximum noise reduction must be applied accompanied by the preservation of the scaffold structure. The result, therefore, is always a compromise between the presence of noise (which reduces pore size) and structure preservation (which in case of reduction leads to the creation of false interconnections between the pores). Micro-CT 2D and 3D analysis is in principle objective. However, we have to consider that the binarization process and data processing prior to image analysis may be substantially influenced by subjectivity.

Micro-CT 3D pore size evaluation is based on sphere fitting algorithm and in principle should be the most precise parameter (direct 3D analysis, whole specimen evaluation, no subjectivity in assessment). 3D analysis of one specimen in high-resolution image datasets took approximately 3 h [Dell Precision T7910, Intel(R) Xeon (R), 3.10 GHz, 3.09 GHz (2 processors), 128 GB RAM], which is substantially more than 2D parameters calculation in 5 slices (~ 10 min). 3D values were approximately 3 times lower than SEM values (“gold standard”), which is important for comparison of studies using SEM or micro-CT analysis.

In order to find convenient simple and time efficient parameter of pore size, we tested 4 parameters based on micro-CT 2D analysis. Major drawbacks are high dependency on pore shape (Fig. 6), image noise and influence of artificial structure defects (e.g. as a result of binarization). Whole specimen evaluation is possible, but is very demanding for further data processing, since 2D evaluation is section-based. There are usually a few thousands slices in each dataset, so usual approach is to limit analysis on certain number of selected slices. Generally, 2D parameters are orientation dependent. Regular circular pores can be easily and time efficiently characterized by these parameters. However, with increasing irregularities, we found important over/underestimations (AECD, MD). Since tissue engineering scaffolds structure is rather complex and irregular, these parameters (AECD, MD) failed in evaluation of pore sizes. Otherwise, the benefits are as mentioned above for micro-CT.

The mean thickness (MT) in 2D is based on circle-fitting and is calculated as an average of all the inscribed circle diameter values (in principle similar to micro-CT 3D), for it is considered to be the most accurate of the micro-CT 2D parameters. MT is sensitive to the presence of noise pixels in pore spaces. The major diameter (MD) considers the furthest distance between two points which can be connected by a straight line within the pore. Higher pore size values can be expected in comparison with the other parameters (Figs. 4, 5). MD is more resistant to image noise (compared to circle-fitting parameters). In the case of non-circular pores (e.g. narrow or lobulated pores), the results generally overestimate the average pore size and may lead to the occurrence of outlier values, as supported by our results. Biggest inner circle diameter (BICD) provides an evaluation of each pore according to the biggest circle which is able to fit the pore. In evaluation of irregularly-shaped pores (e.g. pore with thin prominences), BICD may overestimate pore size. Image noise may significantly reduce BICD values. The BICD and MT pore sizes parameters presented similar values in our study. Area-equivalent circle diameter (AECD) creates a virtual circle of the same area as the pore subjected to analysis and provides an evaluation of the diameter; however, this method leads to substantially higher values in the case of high pore interconnection and artificial structural defects (e.g. due to the partial volume effect and binarization).

### Comparison of different approaches of pore size evaluation

The exact value of pore size is unknown, so all values present some level of uncertainty. For this reason, comparison of different methods based on its accuracy in structural characterization is not achievable. We intended to use coefficient of variation for this purposes, because is a parameter widely used for expressing the repeatability or precision of methods. In our study, comparison of different methods for pore size analysis is problematical because of the nature of these data itself. In general, porosity data evinces high range of scatter. Since we do not compare different approaches of porosity analysis on scaffolds prepared with defined porosity, we are not able to simply explain the variance in data by lower accuracy of applied method. In other words, we are not able to simply conclude whether the method is inaccurate or whether the real porosity truly ranges from units to hundreds of micrometers. Therefore we are not able to quantify the differences between applied methods simply by quantifying the coefficient of variance (Fig. 7). The high variance in data sets of individual methods also invalidate most of the standard statistical tests for the quantification of correlation between each method. Therefore, only qualitative or semi-quantitative evaluation can be performed.

For this reason we decided to use semi-quantitative comparison pore size parameters of relative benefits of eight selected qualities: non-destructivity, time efficacy, orientation independent direct 3D analysis, whole specimen evaluation, high resolution, irregular pore assessment, low image processing bias and widespread use of certain method (described in results in 3.3). We evaluated these qualities in comparison to the other evaluated parameters (Table 2) using following values: 2 (very advantageous), 1 (advantageous), 0 (no advantage). Based on total score calculation, micro-CT 3D analysis was found to be the most convenient method for pore size analysis. Mean thickness parameter was the most convenient in micro-CT 2D analysis and presented the same score as SEM. Other parameters reached lower score. Limitation of this approach is in selection of evaluated qualities and in subjectivity in the assessment.

## Conclusion

Porosity of tissue engineering scaffolds is considered to be their key characteristic. There is no consent on pore size evaluation, which may be obtained using different methods. Since there is no possibility to know the exact pore sizes, comparison of these methods is complicated. In this paper, we focused on comparison SEM (“gold standard”) and micro-CT (emerging imaging method) in pore size evaluation. 6 different pore size parameters (SEM, micro-CT 3D analysis, micro-CT 2D analysis: mean thickness, major diameter, biggest inner circle diameter, area equivalent circle diameter) were applied for the characterization of the pore sizes. Seven model specimens with various 3D structures were prepared and analyzed. Results of pore sizes significantly differed between parameters with median values ranging from 20 to 490 µm. SEM values were approximately three times higher than micro-CT 3D values. Each method and pore size parameter was discussed with its benefits and drawbacks and compared to each other. Comparison of different methods was no applicable using coefficient of variance, so semi-quantitative assessment was applied considering: non-destructivity, time efficacy, orientation independent direct 3D analysis, whole specimen evaluation, high resolution, irregular pore assessment, low image processing bias and widespread use of certain method. We found micro-CT 3D evaluation based on sphere fitting to be the most effective parameter for pore size evaluation. Mean thickness was the most effective micro-CT 2D parameter and may be considered e.g. in evaluation of large sample number evaluation regarding higher time efficacy. Other micro-CT 2D parameters were found to over/underestimate pore size in irregularly shaped pores, which are very frequent in collagen-based scaffolds. SEM is still regarded as gold standard due to its widespread use and high resolution. Comparison and ratio of micro-CT 3D and SEM values is important for understanding studies which are using one or the other approach.

## Additional files

**Additional file 1: Appendix S1.** Cross-section grayscale images of all model specimens: A) 37 MAX B) 37 MID C) 37 MIN D) ORIG E) RT MAX F) RT MID G) RT MIN. Differences in inner structure as a result of different collagen cross-linking procedure is apparent. Scale bar = 500 µm.

**Additional file 2: Appendix S3.** Movie is presenting inner structure of collagen-based scaffold (RT MID specimen). Sphere fitting algorithm is presented as a method applied for pore size evaluation in 3D. Color-coded scale bar is shown on the right.

**Additional file 3: Appendix S2.** Table is presenting pore size values (median, lower and upper quartile; mean and standard deviation; µm) for each specimen and pore size parameter.
