# Supplementary figures and images for: Note on the use of different approaches to determine the pore sizes of tissue engineering scaffolds: what do we measure?
Source: Biomed Eng Online. 2018 Aug 17;17:110. doi: 10.1186/s12938-018-0543-z (PMC6098612; doi:10.1186/s12938-018-0543-z)

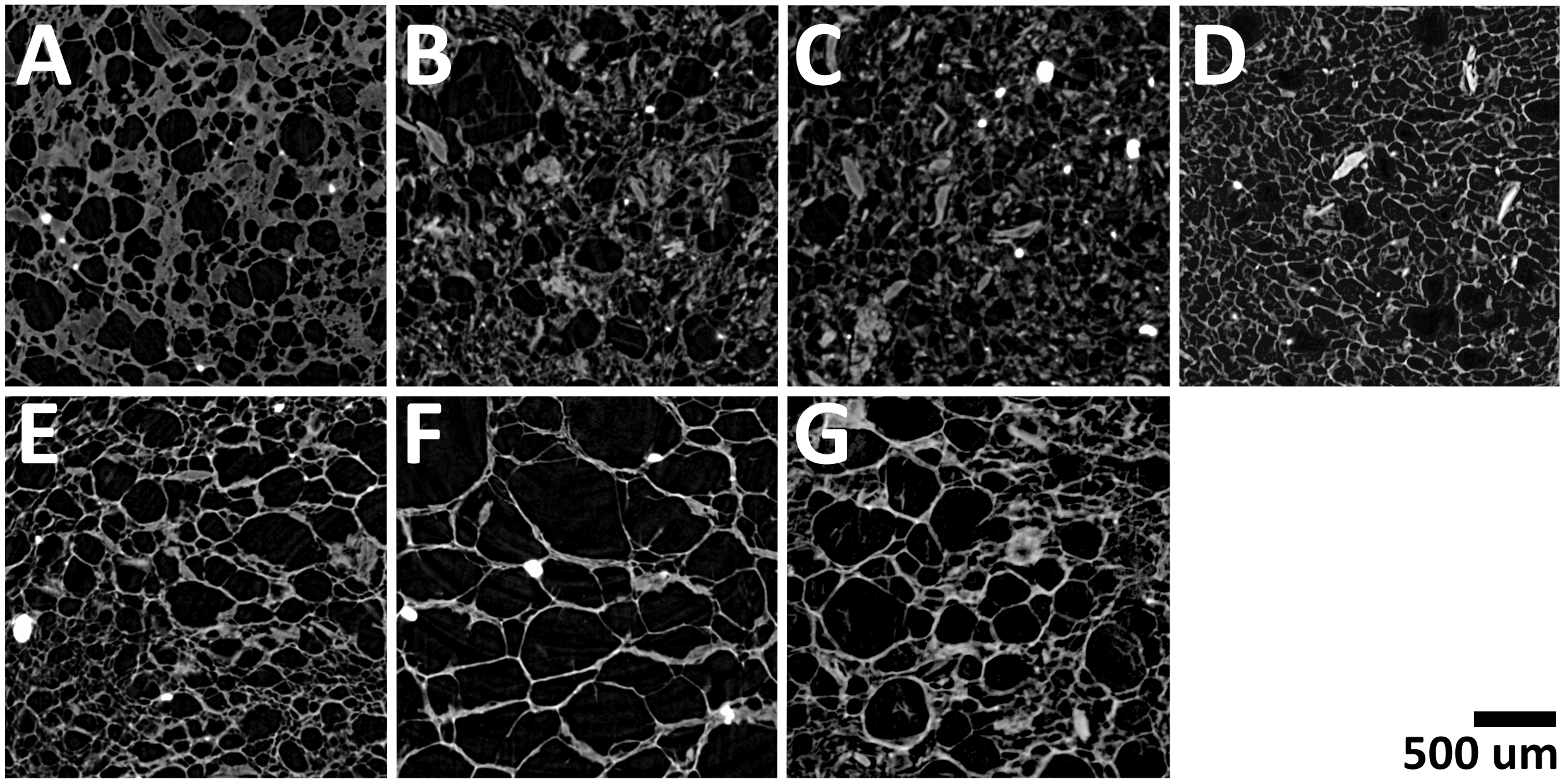

Supplement: Supplementary file 1 — Additional file 1: Appendix S1. Cross-section grayscale images of all model specimens: A) 37 MAX B) 37 MID C) 37 MIN D) ORIG E) RT MAX F) RT MID G) RT MIN. Differences in inner structure as a result of different collagen cross-linking procedure is apparent. Scale bar = 500 µm. [file 12938_2018_543_MOESM1_ESM.tif]
