# Supplementary material for: Note on the use of different approaches to determine the pore sizes of tissue engineering scaffolds: what do we measure?
Source: Biomed Eng Online. 2018 Aug 17;17:110. doi: 10.1186/s12938-018-0543-z (PMC6098612; doi:10.1186/s12938-018-0543-z)
Supplement: Supplementary file 3 — Additional file 3: Appendix S2. Table is presenting pore size values (median, lower and upper quartile; mean and standard deviation; µm) for each specimen and pore size parameter. [file 12938_2018_543_MOESM3_ESM.docx]

|  | **SEM** | | | | | | | **MAJOR DIAMETER** | | | | | | | **MEAN THICKNESS** | | | | | | |
| --- | --- | --- | --- | --- | --- | --- | --- | --- | --- | --- | --- | --- | --- | --- | --- | --- | --- | --- | --- | --- | --- |
|  | **RT MIN** | **RT MID** | **RT MAX** | **37 MIN** | **37 MID** | **37 MAX** | **ORIG** | **RT MIN** | **RT MID** | **RT MAX** | **37 MIN** | **37 MID** | **37 MAX** | **ORIG** | **RT MIN** | **RT MID** | **RT MAX** | **37 MIN** | **37 MID** | **37 MAX** | **ORIG** |
| **MEDIAN** | 171 | 349 | 317 | 93 | 283 | 289 | 147 | 55 | 107 | 70 | 67 | 66 | 97 | 65 | 20 | 32 | 21 | 29 | 28 | 38 | 27 |
| **LOWER QUARTILE** | 124 | 269 | 243 | 56 | 105 | 206 | 109 | 29 | 43 | 37 | 39 | 37 | 47 | 36 | 13 | 15 | 13 | 20 | 20 | 23 | 20 |
| **UPPER QUARTILE** | 373 | 447 | 411 | 144 | 420 | 364 | 196 | 142 | 261 | 158 | 144 | 147 | 201 | 134 | 40 | 72 | 39 | 44 | 45 | 71 | 38 |
| **MEAN** | 239 | 350 | 343 | 158 | 294 | 298 | 156 | 159 | 232 | 140 | 131 | 142 | 149 | 138 | 36 | 51 | 34 | 37 | 38 | 53 | 31 |
| **STANDARD DEVIATION** | 170 | 138 | 147 | 179 | 194 | 129 | 73 | 434 | 356 | 230 | 214 | 230 | 149 | 335 | 43 | 50 | 38 | 27 | 28 | 44 | 18 |

|  | **3D** | | | | | | | **AECD** | | | | | | | **BICD** | | | | | | |
| --- | --- | --- | --- | --- | --- | --- | --- | --- | --- | --- | --- | --- | --- | --- | --- | --- | --- | --- | --- | --- | --- |
|  | **RT MIN** | **RT MID** | **RT MAX** | **37 MIN** | **37 MID** | **37 MAX** | **ORIG** | **RT MIN** | **RT MID** | **RT MAX** | **37 MIN** | **37 MID** | **37 MAX** | **ORIG** | **RT MIN** | **RT MID** | **RT MAX** | **37 MIN** | **37 MID** | **37 MAX** | **ORIG** |
| **MEDIAN** | 72 | 108 | 81 | 72 | 72 | 99 | 45 | 465 | 490 | 365 | 335 | 390 | 240 | 365 | 23 | 37 | 25 | 32 | 32 | 42 | 32 |
| **LOWER QUARTILE** | 45 | 72 | 36 | 45 | 45 | 63 | 36 | 265 | 290 | 190 | 190 | 215 | 165 | 190 | 14 | 16 | 14 | 21 | 21 | 25 | 21 |
| **UPPER QUARTILE** | 126 | 162 | 144 | 117 | 108 | 153 | 72 | 875 | 765 | 640 | 690 | 690 | 365 | 710 | 50 | 95 | 50 | 55 | 55 | 86 | 50 |
| **MEAN** | 90 | 122 | 127 | 88 | 85 | 125 | 79 | 578 | 558 | 456 | 482 | 487 | 309 | 481 | 48 | 69 | 44 | 45 | 47 | 63 | 40 |
| **STANDARD DEVIATION** | 60 | 71 | 165 | 66 | 54 | 96 | 107 | 404 | 337 | 348 | 390 | 353 | 238 | 367 | 64 | 76 | 53 | 43 | 44 | 57 | 34 |
